# Sequential Triple Therapy for Facial Inflammation: Prospective Evaluation of Facial Microneedle, Hydrolifting, and Intense Pulsed Light Therapy

**DOI:** 10.1111/jocd.70327

**Published:** 2025-07-01

**Authors:** Yueling Tang, Bin Liu, Xuefeng Su, Junzheng Wu, Xiaojing Li

**Affiliations:** ^1^ Department of Plastic Surgery The First Affiliated Hospital of Anhui Medical University HeFei China; ^2^ Department of Burn, Plastic and Cosmetic Surgery Xi'an Central Hospital, Xi'an Jiaotong University Xi'an China

**Keywords:** hyaluronic acid, inflammation, intense pulse laser

## Abstract

**Background:**

Facial inflammation is a highly prevalent condition mediated by diverse etiologies, primarily manifested as facial redness, swelling, itching, pain, maculopapular rash, or even pustules. Conventional treatments such as topical or oral medications for anti‐inflammatory, antibacterial, and immunomodulatory purposes have shown limitations, particularly in the slow resolution of symptoms. While standalone laser therapy or hydrolifting may provide relatively faster symptom relief, their efficacy remains insufficiently stable or durable.

**Objective:**

This prospective, comparative, and randomized clinical study aims to investigate the therapeutic effects of combining facial microneedling with intense pulse laser (IPL) and hydrolifting for facial inflammation.

**Methods:**

From January 2022 to January 2024, 114 patients with facial inflammation were enrolled and randomly allocated into one experimental group and two control groups. The experimental group received triple therapy, while the control groups received either hydrolifting alone or IPL monotherapy. Therapeutic outcomes were evaluated by comparing improvements in inflammatory area reduction, aesthetic indicators, Global Aesthetic Improvement Scale (GAIS) scores, and incidence of adverse events post‐treatment.

**Results:**

Baseline characteristics showed no significant differences among the three groups before treatment. At 2 weeks, 3 months, and 6 months post‐treatment, the experimental group demonstrated significantly better outcomes in inflammatory area reduction and GAIS scores compared to the control groups. Although aesthetic indicators improved in all groups, intergroup differences were not statistically significant. No significant differences in adverse event rates were observed among the groups.

**Conclusion:**

This prospective, controlled, and randomized clinical study demonstrates that microneedling‐assisted IPL combined with hydrolifting yields superior and more sustained therapeutic efficacy in facial anti‐inflammatory treatment.

## 1 Introduction

Facial inflammation is a highly prevalent global disorder characterized by pathological reactions in the skin and subcutaneous tissues mediated by diverse etiological factors [1, 2, 3]. Its clinical manifestations vary depending on the underlying causes and disease progression stages. Acute facial inflammation typically presents with erythema, edema, papules, pustules, and symptoms of pruritus or pain [4, 5, 6]. In chronic cases, patients may develop scaling or lichenification [7], while systemic diseases can lead to additional systemic symptoms such as fever and arthralgia [8]. The etiology of facial inflammation is complex and often involves multifactorial interactions, including microbial infections, environmental irritants, endocrine and metabolic dysregulation, and aberrant immune responses [9, 10, 11].

Conventional therapeutic approaches for facial inflammation primarily focus on anti‐inflammatory, antimicrobial, immunomodulatory, and physical interventions [12]. These include topical corticosteroids, antibiotic or antifungal ointments, retinoids, oral antibiotics, immunosuppressants, isotretinoin, as well as physical modalities such as phototherapy and cryotherapy. Despite their widespread clinical use, these traditional treatments exhibit significant limitations, including antibiotic resistance, steroid dependency, skin barrier impairment, and systemic toxicity associated with retinoids, transient efficacy, high recurrence rates, and poor patient compliance due to complex regimens [13, 14].

Notably, in our clinical practice, a substantial proportion of patients with facial inflammation attribute their condition to environmental irritants or hormonal imbalances, with primary complaints centered on resolving localized symptoms such as erythema and swelling. To address these concerns, we have increasingly adopted minimally invasive techniques, including hydrolifting and laser therapies. Over extended clinical observation, these modalities have demonstrated superior safety, efficacy, and long‐term outcomes compared to conventional approaches. Photoelectric therapies enable comprehensive skin rejuvenation through photomodulation [15], while hydrolifting facilitates targeted delivery of nutrients or reparative agents into the dermis [16].

Building on these observations, we hypothesized that combining photoelectric therapy with hydrolifting, augmented by microneedling to enhance penetration and stimulate collagen remodeling, could synergistically improve therapeutic outcomes for facial inflammation. To validate this hypothesis, we conducted a prospective randomized controlled study to evaluate the efficacy and safety of microneedling‐assisted combined photoelectric and hydrolifting therapy in managing facial inflammatory conditions.

## 2 Patients and Methods

This study was conducted in accordance with the Declaration of Helsinki and approved by the hospital ethics committee, with written informed consent obtained from all participants. The inclusion criteria were as follows: (1) age between 18 and 65 years, regardless of gender; (2) Fitzpatrick skin phototypes I–IV; (3) clinical diagnosis of facial inflammatory conditions characterized by erythema, edema, pruritus, pain, and enlarged pores, with etiological factors attributable to environmental physicochemical factors or endogenous hormonal metabolic abnormalities; and (4) voluntary provision of informed consent. Exclusion criteria included: (1) facial inflammation caused by infectious or immune‐related disorders such as acute phase of rosacea or facial Demodex mite infestation, or other conditions contraindicating the proposed treatment; (2) history of facial laser therapy or injectable treatments within the preceding 6 months; and (3) withdrawal from the study prior to completion. Using a random number table, 114 patients were allocated to three groups: Group A (experimental) received microneedling‐assisted combined photoelectric therapy and hydrolifting, while Groups B (photoelectric therapy alone) and C (hydrolifting alone) served as controls.

### 2.1 Sample Size Calculation

Based on pilot study data, the sample size was determined using PASS software, with the inflammatory area at 2 weeks post‐treatment as the primary outcome. Assuming means of 0.03, 0.06, and 0.07 for Groups A, B, and C, respectively, a common standard deviation of 0.045, a type I error (*α*) of 0.05, and a type II error (*β*) of 0.10 (90% power), the calculated sample size was 31 participants per group. To account for a potential 20% attrition rate, 38 participants were ultimately enrolled in each group.

### 2.2 Surgical Technique

All patients underwent pre‐treatment evaluation using the VISIA Complexion Analysis System, with deep red areas in specific modes indicating capillary dilation and inflammation. Facial inflammation severity was classified as mild, moderate, or severe based on the extent of these regions (ImageJ software was used to calibrate grayscale thresholds for precise segmentation of inflammatory regions. The validated threshold range was applied to both longitudinal analyses [same individual across timepoints] and cross‐sectional comparisons [inflammation area percentage between individuals]). For Group A (experimental), treatment comprised two phases per cycle: microneedling therapy followed 1 month later by combined photoelectric therapy and hydrolifting, with three full cycles completed over 6 months. Group B received photoelectric therapy alone, involving five sessions administered monthly for the first three treatments and every 1.5 months for the remaining two, totaling 6 months. Similarly, Group C underwent hydrolifting alone following the same fivesession schedule as Group B. All procedures were standardized and performed by a single physician to ensure consistency.

#### 2.2.1 Facial Microneedle

**FIGURE 1:**
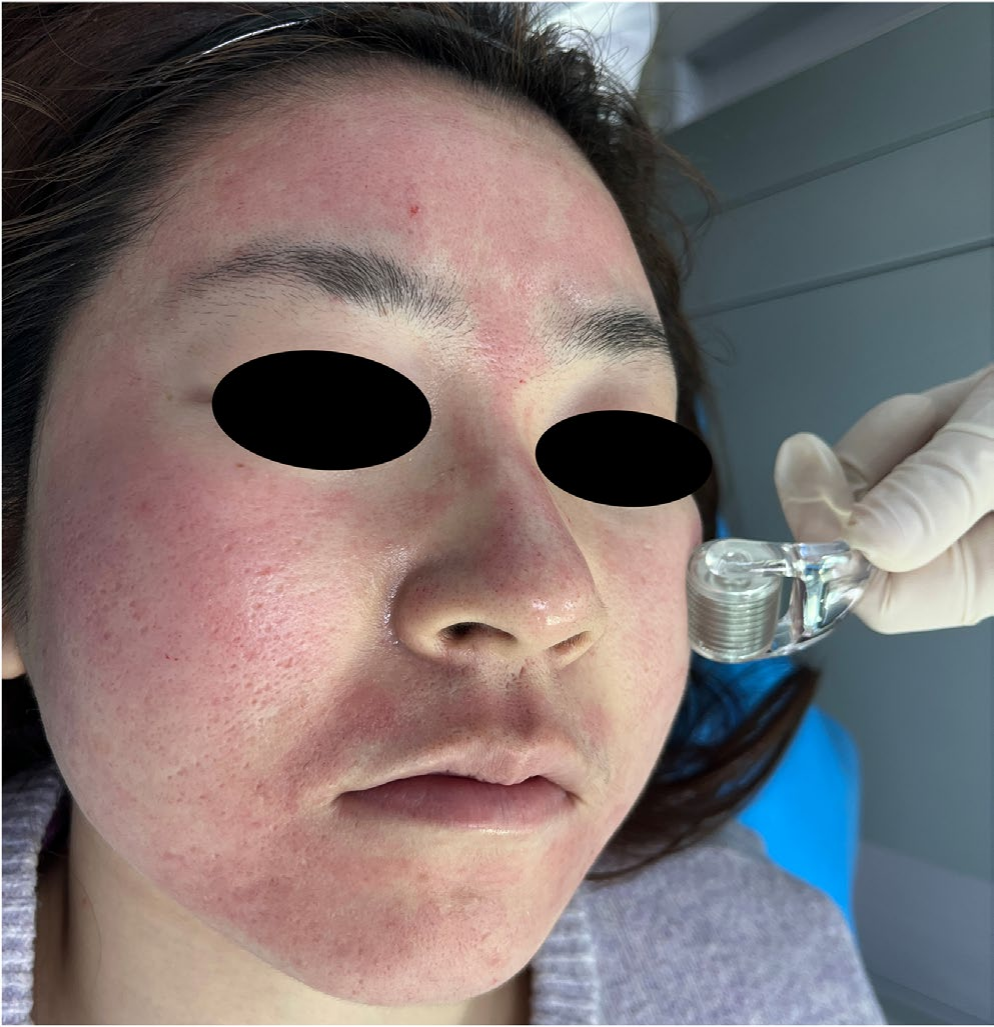
Facial microneedling treatment.

Prior to the procedure, skin was cleansed with a soap‐free facial cleanser to remove sebum and cosmetic residues, followed by topical application of compound lidocaine cream for 30 min for local anesthesia. Routine disinfection with iodophor and alcohol deiodination was performed. A 0.5 mm microneedling roller was used, with the physician holding the device at a 45° angle and rolling multidirectionally along facial muscle lines (horizontally on the forehead, obliquely upward and outward on the cheeks, and vertically on the mandible). Uniform pressure was applied to ensure vertical penetration of microneedles. Each facial region was rolled 8–10 times until uniform pinpoint bleeding occurred, confirming dermal microchannel formation (Figure 1). Subsequently, a mucoprotein wound repair dressing (FORSMILE; Yibei, China) was applied, followed by a wet compress with 40 U botulinum toxin (Botox, Allergan, Sweden) for 15 min and coverage with a medical‐grade hydrating mask. Post‐treatment care included daily saline cleansing for 3 days, application of medical‐grade reparative moisturizers, and strict adherence to sun protection measures.

#### 2.2.2 IPL Therapy

The IPL system (Queen, Wuhan, China) was employed for treatment. Patients first cleansed their skin with a soap‐free facial cleanser to remove sebum and cosmetic residues. Hair was covered with a disposable surgical cap, and patients were positioned supine with eyes protected by double‐layered gauze or goggles. A 2–3 mm thick medical coupling gel was uniformly applied to the treatment area. Parameters were adjusted based on inflammation severity: mild cases received a 560 nm wavelength with dual pulse widths of 2.8 ms/3.6 ms, 25 ms pulse delay, and 16–20 J/cm^2^ energy density; moderate cases used 560 nm, 3.0 ms/3.8 ms pulse widths, 28 ms delay, and 14–18 J/cm^2^; severe cases were treated with 560 nm, 4.0–4.5 ms pulse widths, 30 ms delay, and 10–14 J/cm^2^. The IPL handpiece was held perpendicular to the skin, scanning sequentially from forehead to cheeks, nose, and mandible without spot overlap, with repeated passes over severely inflamed areas. Each session lasted 20–30 min. Post‐treatment care included daily 15–20 min cold compresses with medical‐grade hydrating masks for 7 days and strict sun protection.

#### 2.2.3 Hydrolifting

Prior to treatment, facial skin was cleansed, followed by topical application of compound lidocaine cream under plastic wrap occlusion for 60 min. After removal and disinfection, the Skin2 hydrolifting device (Hoya Beauty, Shanghai, China) was equipped with a sterile 9‐port needle (3 × 3 configuration) and set to “Dose very fast” mode with 20% negative pressure. A sodium hyaluronate complex solution (hyaluronic acid vital; Imeik) was injected at a depth of 0.85 mm, with a per‐point dosage of 0.0179–0.0208 mL. Total administered volumes were adjusted based on inflammation severity: 2 mL for mild, 3 mL for moderate, and 5 mL for severe cases. The solution was delivered sequentially to non‐overlapping zones of the upper, middle, and lower face until complete depletion. Post‐procedure care included a 20‐min cold compress with a 4°C medical‐grade hydrating mask, daily application of reparative moisturizers for 7 days, and strict sun protection.

### 2.3 Item Assessment

The evaluated parameters encompassed: (1) baseline demographics (age, gender, lifestyle habits including smoking/alcohol use), pre‐treatment inflammatory area, and inflammation severity categorized as mild (< 10% facial involvement), moderate (10%–20%), or severe (> 20%), alongside Self‐Rating Anxiety Scale [17] (SAS) scores (normal: < 50; exclusion threshold: ≥ 70); (2) inflammatory area reduction at 2 weeks, 3 months, and 6 months post‐treatment; (3) aesthetic outcomes including skin texture (graded by roughness [mild: 20–25 μm, moderate: 25–30 μm, severe: > 30 μm] and smoothness [mild: 70–80, moderate: 60–70, severe: < 60]) and pore size (mild: 0.1–0.2 mm diameter/5%–7% area, moderate: 0.2–0.3 mm/7%–10%, severe: > 0.3 mm/> 10%); (4) Global Aesthetic Improvement Scale [18] (GAIS) scores (−1 [worsened] to 3 [complete improvement]) from both observers and patients; (5) post‐procedural pain assessed via Visual Analogue Scale [19] (VAS: 0 [none] to 10 [severe]); and (6) adverse event rates across groups.

### 2.4 Statistic Analysis

Data processing and statistical analyses were performed using R software (version 4.3.2). Normally distributed continuous variables were expressed as mean ± standard deviation and compared across groups using ANOVA with post hoc least significant difference (LSD) tests. Non‐normally distributed continuous variables were reported as median (interquartile range) [*M* (*Q*1, *Q*3)] and analyzed via Kruskal–Wallis *H* tests, followed by Bonferroni‐corrected pairwise comparisons. Categorical variables were presented as counts (percentages) [*n* (%)] and evaluated using chi‐squared tests.

Generalized estimating equations (GEE) with an exchangeable working correlation structure were employed to analyze longitudinal changes in inflammatory area. The model included independent variables: group assignment, follow‐up time points (categorical), group–time interaction, and baseline inflammatory area (continuous). Age and sex were adjusted to further examine the association between group allocation and inflammatory outcomes. All statistical tests were two‐sided, with a significance level set at *α* = 0.05.

## 3 Results

**FIGURE 2:**
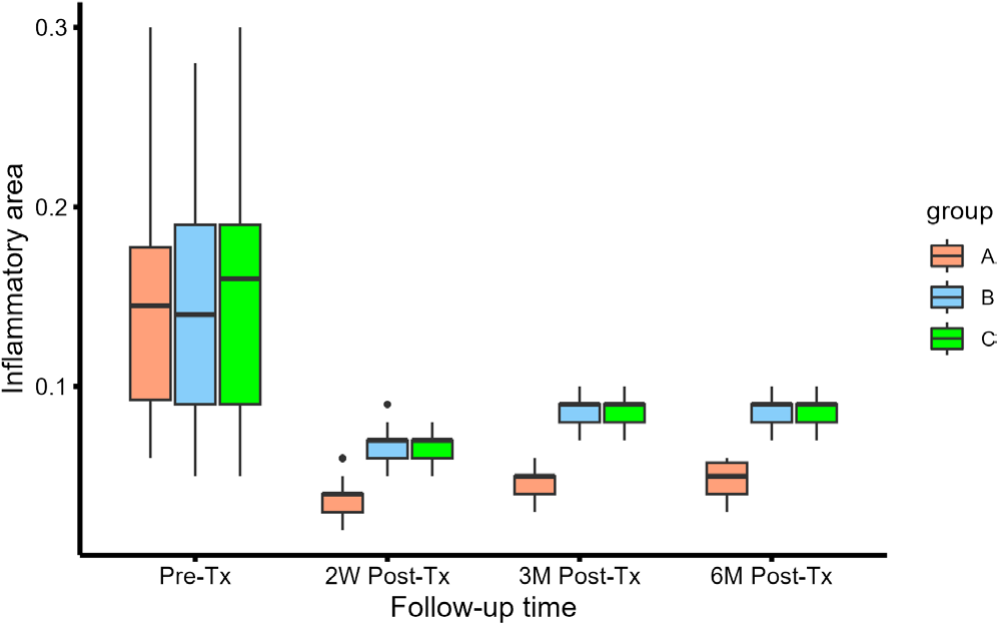
Generalized equation analysis demonstrates that inflammatory area scores in Groups B and C significantly increased over time compared to Group A, confirming the superior long‐term efficacy of the experimental triple‐combination therapy.

From January 2022 to January 2024, 114 patients (100 females, 14 males; mean age 24.04 ± 3.03 years) were enrolled, with no significant baseline differences among groups in demographics, lifestyle factors, pre‐treatment inflammation severity, or skin/pore characteristics (*p* > 0.05; Table 1). Group A (microneedling‐assisted combined therapy) demonstrated superior reductions in inflammatory area compared to Groups B (photoelectric therapy alone) and C (hydrolifting alone) at 2 weeks, 3 months, and 6 months (*p* < 0.05), while no differences existed between B and C (Table 2). All groups showed comparable improvements in skin roughness, smoothness, and pore size (Table 3). Observer‐ and patient‐rated GAIS scores significantly favored Group A over both control groups (*p* < 0.001), though no intergroup differences in pain scores (VAS < 4) or adverse event rates (erythema, edema, hyperpigmentation) were observed (Tables 4 and 5). Generalized estimating equations revealed significantly increasing inflammatory scores over time in Groups B (*β* = 0.0284, 95% CI: 0.0236–0.0412; *p* < 0.001) and C (*β* = 0.0276, 95% CI: 0.0230–0.0322; *p* < 0.001) compared to Group A, adjusted for baseline values, age, and sex (Figure 2).

**TABLE 1:** Baseline demographic characteristics.

Variables	Group A	Group B	Group C	Statistical value	*p*
Age	33.76 ± 3.11	34.24 ± 3.11	34.13 ± 2.87	0.256	0.775
Inflammatory area	0.15 (0.09, 0.18)	0.14 (0.09, 0.19)	0.16 (0.09, 0.19)	0.11	0.947
Roughness	26 (24, 28)	26 (24, 27)	25 (24, 28)	1.39	0.499
Smoothness	69.11 ± 6.62	68.37 ± 7.56	67.24 ± 4.68	1.067	0.35
Facial pore diameter	0.24 ± 0.07	0.23 ± 0.08	0.26 ± 0.08	1.104	0.335
Pore area percentage	8 (6.42, 9.52)	7.8 (6.65, 9.6)	7.9 (6.32, 9.47)	0.087	0.957
SAS score	48.24 ± 9.37	47.24 ± 10.96	44.87 ± 9.49	1.145	0.322
Gender				Fisher	1
Male	5 (13.16)	5 (13.16)	4 (10.53)		
Female	33 (86.84)	33 (86.84)	34 (89.47)		
Smoking				Fisher	1
No	34 (89.47)	34 (89.47)	34 (89.47)		
Yes	4 (10.53)	4 (10.53)	4 (10.53)		
Drinking status				0.717	0.699
No	28 (73.68)	30 (78.95)	31 (81.58)		
Yes	10 (26.32)	8 (21.05)	7 (18.42)		
Inflammation severity				0.282	0.991
Mild	10 (26.32)	11 (28.95)	11 (28.95)		
Moderate	20 (52.63)	18 (47.37)	18 (47.37)		
Severe	8 (21.05)	9 (23.68)	9 (23.68)		
Skin texture				3.538	0.472
Mild	5 (13.16)	6 (15.79)	4 (10.53)		
Moderate	27 (71.05)	24 (63.16)	31 (81.58)		
Severe	6 (15.79)	8 (21.05)	3 (7.89)		
Pore grading				Fisher	0.952
Mild	2 (5.26)	4 (10.53)	3 (7.89)		
Moderate	22 (57.89)	20 (52.63)	20 (52.63)		
Severe	14 (36.84)	14 (36.84)	15 (39.47)		

**TABLE 2:** Comparison of inflammatory area.

	Group A	Group B	Group C	Generalized estimation equation model			
				Unadjusted intervention effect		Adjusted intervention effect^a^	
				*β* (95% CI)	*p*	*β* (95% CI)	*p*
Area				0.0284 (0.0236, 0.0412)^b^	< 0.001	0.0285 (0.0237, 0.0333)^b^	< 0.001
Baseline	0.15 (0.067)	0.148 (0.066)	0.152 (0.069)	0.0276 (0.0230, 0.0322)^c^	< 0.001	0.0277 (0.0231, 0.0322)^c^	< 0.001
2 Weeks post	0.037 (0.011)	0.066 (0.01)	0.065 (0.009)				
3 Months post	0.048 (0.009)	0.086 (0.009)	0.088 (0.008)				
6 Months post	0.048 (0.009)	0.086 (0.009)	0.087 (0.009)				

^a^ Adjusted for age and gender.

^b^ Denotes comparison between Group B and Group A.

^c^ Denotes comparison between Group C and Group A.

**TABLE 3:** Comparison of facial aesthetic indicator.

Variables	Time	Group A	Group B	Group C	Statistical value	*p*
Roughness	2 weeks post	23.41 ± 3.21	23.93 ± 2.94	24.3 ± 2.92	0.821	0.443
	3 months post	24.34 ± 3.35	24.41 ± 2.95	24.73 ± 2.95	0.17	0.844
	6 months post	25.44 (23.02, 27.53)	25.59 (23.28, 26.56)	24.7 (23.19, 27.26)	0.187	0.911
Smoothness	2 weeks post	79.32 ± 6.61	77.42 ± 7.82	76.32 ± 5.06	2.438	0.095
	3 months post	76.02 ± 6.37	74.36 ± 7.77	73.33 ± 4.92	2.109	0.129
	6 months post	72.48 ± 6.65	71.33 ± 7.71	70.16 ± 4.88	1.518	0.226
Facial pore diameter	2 weeks post	0.22 ± 0.07	0.22 ± 0.08	0.24 ± 0.07	1.586	0.209
	3 months post	0.23 ± 0.07	0.22 ± 0.08	0.25 ± 0.07	1.275	0.283
	6 months post	0.23 ± 0.07	0.23 ± 0.08	0.25 ± 0.07	1.179	0.311
Pore area percentage	2 weeks post	7.66 (6.27, 9.14)	7.65 (6.43, 9.51)	7.72 (6.22, 9.35)	0.306	0.858
	3 months post	7.77 (6.34, 9.32)	7.7 (6.49, 9.54)	7.78 (6.28, 9.37)	0.188	0.91
	6 months post	7.86 (6.39, 9.41)	7.71 (6.57, 9.56)	7.84 (6.3, 9.41)	0.112	0.946
Skin texture	2 weeks post				Fisher	0.338
	Mild	23 (60.53)	18 (47.37)	20 (52.63)		
	Moderate	15 (39.47)	20 (52.63)	16 (42.11)		
	Severe	0 (0)	0 (0)	2 (5.26)		
	3 months post				Fisher	0.658
	Mild	18 (47.37)	12 (31.58)	17 (44.74)		
	Moderate	18 (47.37)	24 (63.16)	19 (50)		
	Severe	2 (5.26)	2 (5.26)	2 (5.26)		
	6 months post				Fisher	0.764
	Mild	8 (21.05)	7 (18.42)	11 (28.95)		
	Moderate	25 (65.79)	28 (73.68)	24 (63.16)		
	Severe	5 (13.16)	3 (7.89)	3 (7.89)		
Pore grading	2 weeks post				2.274	0.685
	Mild	7 (18.42)	6 (15.79)	3 (7.89)		
	Moderate	20 (52.63)	19 (50)	20 (52.63)		
	Severe	11 (28.95)	13 (34.21)	15 (39.47)		
	3 months post				1.679	0.794
	Mild	6 (15.79)	6 (15.79)	3 (7.89)		
	Moderate	20 (52.63)	18 (47.37)	20 (52.63)		
	Severe	12 (31.58)	14 (36.84)	15 (39.47)		
	6 months post				Fisher	0.942
	Mild	5 (13.16)	5 (13.16)	3 (7.89)		
	Moderate	20 (52.63)	19 (50)	20 (52.63)		
	Severe	13 (34.21)	14 (36.84)	15 (39.47)		

**TABLE 4:** GAIS and VAS score.

Variables	Group A	Group B	Group C	Statistical value	*p*
GAIS‐observer	2 (2, 3)	1 (1, 2)^a^	1 (0, 2)^a^	31.093	< 0.001
GAIS‐patient	2 (2, 3)	1 (1, 2)^a^	1 (1, 2)^a^	30.777	< 0.001
VAS	2 (2, 3)	2 (1, 3)	2 (1, 3)	0.602	0.74

^a^ Indicates a statistically significant difference compared to Group A.

**TABLE 5:** Complications post‐treatment.

Complications	Group A	Group B	Group C	Statistical value	*p*
Erythema				Fisher	0.703
No	35 (92.11)	35 (92.11)	37 (97.37)		
Yes	3 (7.89)	3 (7.89)	1 (2.63)		
Swelling				Fisher	0.882
No	35 (92.11)	36 (94.74)	37 (97.37)		
Yes	3 (7.89)	2 (5.26)	1 (2.63)		
Infection				Fisher	1
No	37 (97.37)	38 (100)	38 (100)		
Yes	1 (2.63)	0 (0)	0 (0)		
Hyper‐pigmentation				Fisher	0.757
No	36 (94.74)	37 (97.37)	38 (100)		
Yes	2 (5.26)	1 (2.63)	0 (0)		

## 4 Cases Report

### 4.1 Case 1

**FIGURE 3:**
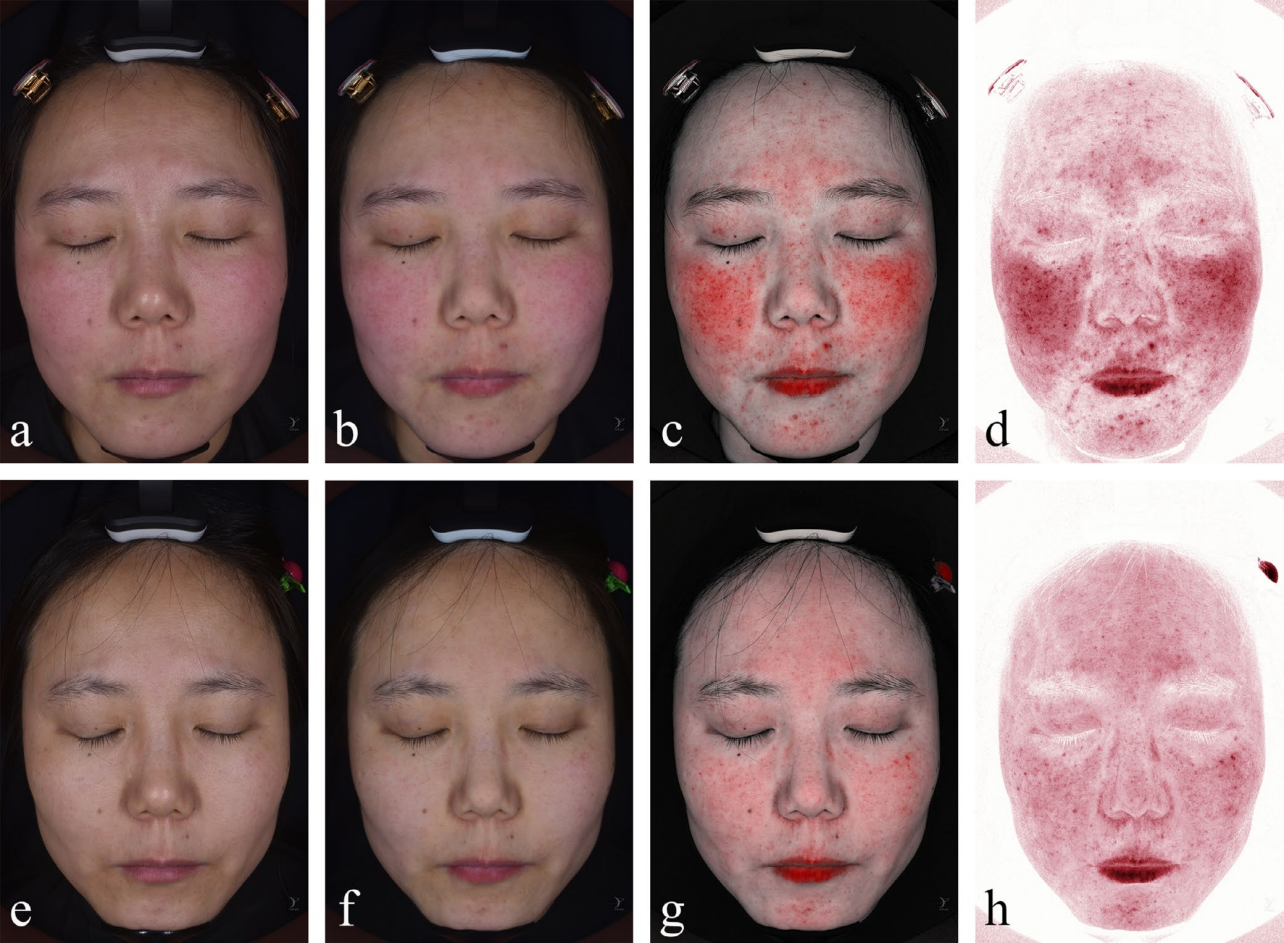
(a–d) Natural light imaging (pre‐treatment), cross‐polarized imaging (for evaluating pores and skin texture), near‐infrared imaging, and combined infrared imaging (deep red areas excluding the lips indicate facial inflammatory regions) before treatment. (e–h) Natural light imaging, cross‐polarized imaging, near‐infrared imaging, and combined infrared imaging after treatment.

A 31‐year‐old female patient in Group A presented with facial inflammation localized predominantly in the midface region, attributed to hypersensitive skin and hormonal dysregulation. Pre‐treatment VISIA analysis revealed a red area ratio of 23.76%, classified as severe facial inflammation. Following the microneedling‐assisted combined therapy protocol, the inflammatory area significantly decreased to 14.54%, with the patient reporting high satisfaction regarding both clinical outcomes and aesthetic improvement (Figure 3).

### 4.2 Case 2

**FIGURE 4:**
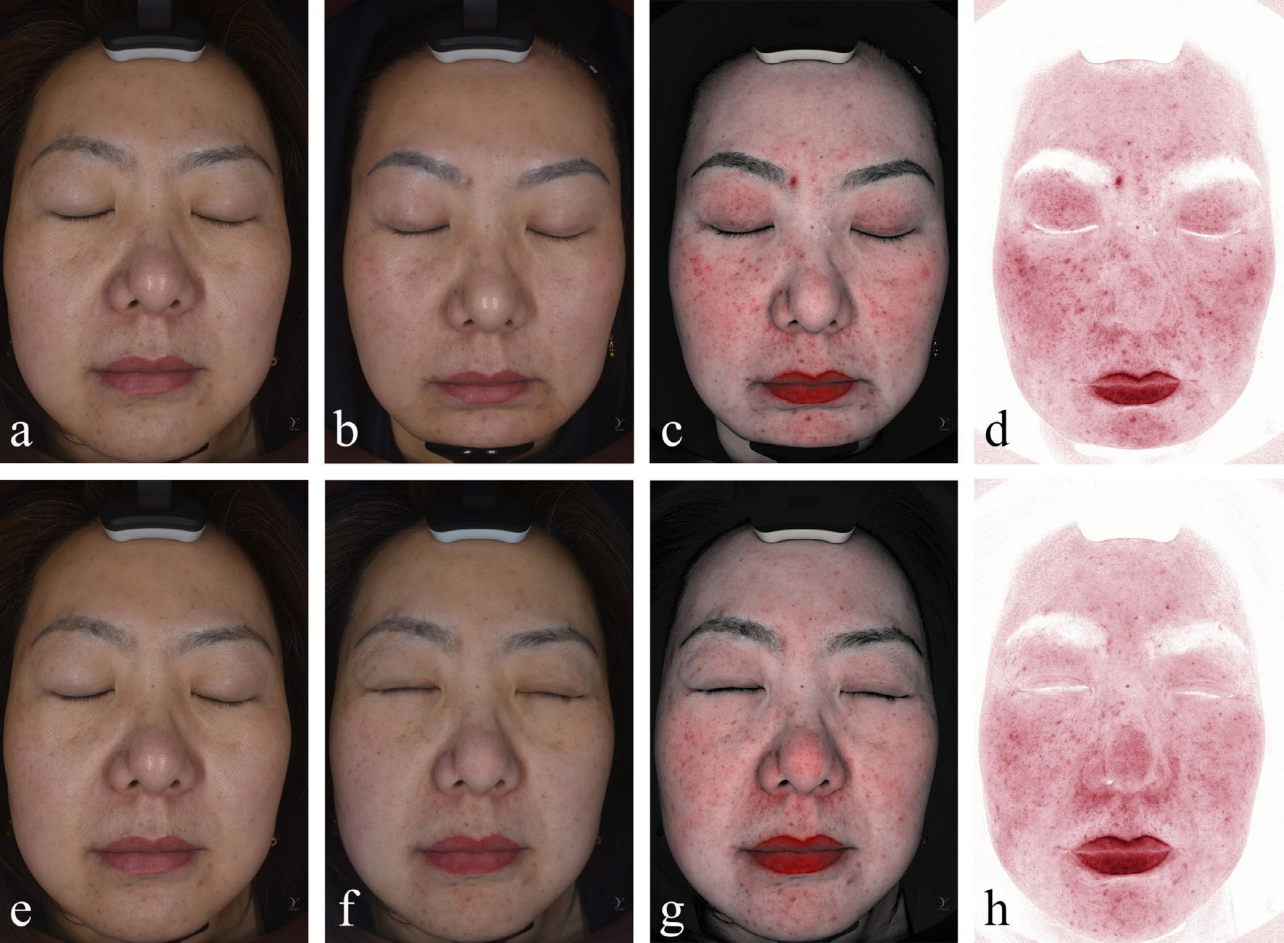
(a–d) Natural light imaging (pre‐treatment), cross‐polarized imaging (for evaluating pores and skin texture), near‐infrared imaging, and combined infrared imaging (deep red areas excluding the lips indicate facial inflammatory regions) before treatment. (e–h) Natural light imaging, cross‐polarized imaging, near‐infrared imaging, and combined infrared imaging after treatment.

A 35‐year‐old female patient in Group B presented with hormonally driven facial acne and inflammation distributed across the midface and mandibular regions. Pre‐treatment VISIA assessment identified a red area ratio of 13.56%, categorized as moderate facial inflammation. Following photoelectric therapy alone, the inflammatory area decreased to 9.27%, accompanied by visible improvement in pore‐related concerns (Figure 4).

### 4.3 Case 3

**FIGURE 5:**
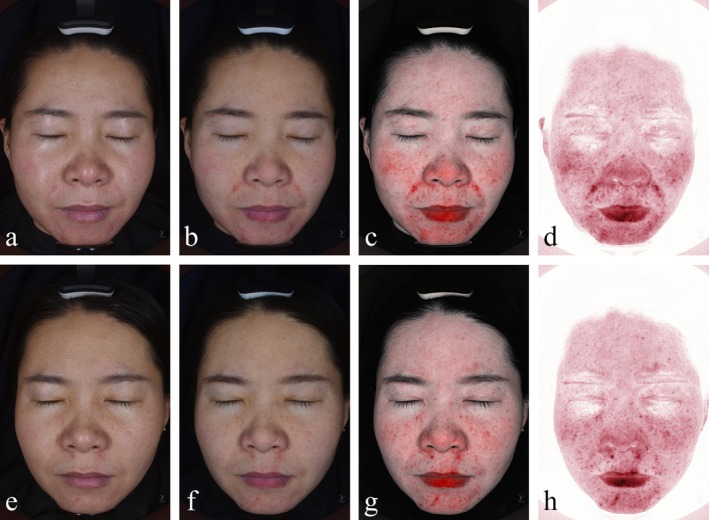
(a–d) Natural light imaging (pre‐treatment), cross‐polarized imaging (for evaluating pores and skin texture), near‐infrared imaging, and combined infrared imaging (deep red areas excluding the lips indicate facial inflammatory regions) before treatment. (e–h) Natural light imaging, cross‐polarized imaging, near‐infrared imaging, and combined infrared imaging after treatment.

A 33‐year‐old female patient in Group C exhibited hormonally or metabolically driven facial acne with inflammatory lesions concentrated in the mid‐to‐lower face, particularly periorally. Pre‐treatment VISIA evaluation demonstrated a red area ratio of 21.62%, classified as severe facial inflammation. Post‐hydrolifting therapy alone, the inflammatory area showed a modest reduction to 18.86%, with concurrent improvements in pore appearance and skin texture (Figure 5).

## 5 Discussion

In contemporary society, appearance‐related anxiety has become a prevalent psychological concern, particularly among patients with facial inflammation [20]. Persistent erythema, papules, pustules, and other lesions not only cause physical discomfort but also contribute to social withdrawal and diminished self‐esteem. As dermatologists, we recognize patients' dual demands: rapid resolution of visible symptoms and long‐term restoration of a stable skin barrier. This motivated our development of a triple‐combination therapy for facial inflammatory conditions. Previous studies primarily focused on single‐modality anti‐inflammatory mechanisms. For instance, laser therapy enhances cellular metabolism and ATP synthesis by oxidizing cytochrome *c* oxidase, promotes nitric oxide release for vasodilation, and modulates key inflammatory mediators (e.g., nuclear factor kappa B, NF‐κB), thereby suppressing prostaglandin E2 (PGE2), tumor necrosis factor‐alpha (TNF‐α), cyclooxygenase‐2 (COX‐2), and interleukin‐1β (IL‐1β) [21, 22]. Similarly, hyaluronic acid (HA) interacts with cell surface receptors (CD44, TLR2/4, RHAMM) to regulate inflammatory pathways (NF‐κB, MAPK), inhibit pro‐inflammatory cytokines (TNF‐α, IL‐6, IL‐1β), and promote macrophage polarization and tissue repair [23].

While these monotherapies demonstrate partial efficacy, their limitations—such as incomplete anti‐inflammatory effects and recurrence within 6 months—highlight the need for synergistic approaches. Our generalized estimating equation analysis revealed widening therapeutic disparities among groups over time (Figure 4), underscoring the superior durability and potency of the combined regimen. Mechanistically, microneedling‐induced mechanical stress disrupts the epidermal barrier, creating transient microchannels that enhance drug permeability and sensitize inflamed tissues to photoelectric energy [24]. Concurrently, microneedling stimulates fibroblast proliferation, collagen/elastin synthesis, and dermal remodeling [25]. Synergy with IPL ensures precise photon delivery to inflammatory foci, while hydrolifting prolongs the retention of reparative agents, collectively achieving comprehensive anti‐inflammatory, barrier‐restorative, and regenerative outcomes.

Our study has limitations. First, the lack of mechanistic investigations precludes definitive conclusions about the molecular drivers of the observed synergy. Second, the single‐center design necessitates validation through multicenter trials to enhance generalizability. Future research should integrate multi‐omics analyses to unravel cellular and molecular interactions underlying this combinatorial efficacy.

## 6 Conclusion

Through this prospective, controlled, and randomized clinical study, we have demonstrated that the combined regimen of microneedling‐assisted IPL and hydrolifting exhibits superior and more sustained therapeutic efficacy in managing facial inflammatory conditions compared to monotherapy approaches. This synergistic strategy not only addresses acute inflammatory manifestations but also promotes long‐term skin barrier restoration, offering a clinically viable solution for patients seeking comprehensive and durable outcomes.

## Author Contributions

Y.T. and B.L. performed the research. X.L. and X.S. supervised the research study. J.W. and Y.T. analyzed the data. Y.T. wrote the paper.

## Conflicts of Interest

The authors declare no conflicts of interest.

## Data Availability

The data that support the findings of this study are available from the corresponding author upon reasonable request.
